# Reconstruction of Complex Directional Networks with Group Lasso Nonlinear Conditional Granger Causality

**DOI:** 10.1038/s41598-017-02762-5

**Published:** 2017-06-07

**Authors:** Guanxue Yang, Lin Wang, Xiaofan Wang

**Affiliations:** Department of Automation, Shanghai Jiao Tong University, and Key Laboratory of System Control and Information Processing, Ministry of Education of China, Shanghai, 200240 P. R. China

## Abstract

Reconstruction of networks underlying complex systems is one of the most crucial problems in many areas of engineering and science. In this paper, rather than identifying parameters of complex systems governed by pre-defined models or taking some polynomial and rational functions as a prior information for subsequent model selection, we put forward a general framework for nonlinear causal network reconstruction from time-series with limited observations. With obtaining multi-source datasets based on the data-fusion strategy, we propose a novel method to handle nonlinearity and directionality of complex networked systems, namely group lasso nonlinear conditional granger causality. Specially, our method can exploit different sets of radial basis functions to approximate the nonlinear interactions between each pair of nodes and integrate sparsity into grouped variables selection. The performance characteristic of our approach is firstly assessed with two types of simulated datasets from nonlinear vector autoregressive model and nonlinear dynamic models, and then verified based on the benchmark datasets from DREAM3 Challenge4. Effects of data size and noise intensity are also discussed. All of the results demonstrate that the proposed method performs better in terms of higher area under precision-recall curve.

## Introduction

In recent years, there has been an explosion of various datasets, which are collected in scientific, engineering, medical and social applications^1, 2^. They often contain information that represents a combination of different properties of the real world. Identifying causality or correlation among datasets^3–7^ is increasingly vital for effective policy and management recommendations on climate, epidemiology, neuroscience, economics and much else. With these rapid advances in the studies of causality and correlation, complex network reconstruction has become an outstanding and significant problem in interdisciplinary science^8–12^. As we know, numerous real networked systems could be represented as network of interconnected nodes. But in lots of situations, network topology is fully unknown, which is hidden in the observations acquired from experiments. For complex systems, accompanied by the complexity of system dynamics, the limited observations with noisy measurements make the problem of network reconstruction even more challenging. An increased attention for network reconstruction is being attracted in the past few years^13–19^.

Among the developed methods, vector autoregressive model (VAR) is able to estimate the temporal dependencies of variables in multivariate model, which gains growing interest in recent years^20–22^. As one of the most prevalent VAR methods, Granger Causality (GC) can be efficiently applied in causal discovery^23^. Conditional Granger Causality (CGC) is put forward to differentiate direct interactions from indirect ones^24^. To extend the application of GC limited by linear dynamics, nonlinear GC is developed^25–28^, which is relatively less considered until now. Generally speaking, the application of these GC methods might get in trouble, especially when the size of samples is small and the number of variables is large. To conquer such problem, some composed methods are presented by integrating variable selection into CGC model, such as Lasso-CGC^29, 30^ and grouped lasso graphical granger^31^. Group lasso is also used in multivariate regression and multi-task learning^32–36^. Compressive sensing or sparse regression is popularly applied in the network reconstruction and system identification. However, most of these methods are confined to identify parameters of complex systems governed by pre-defined models^18, 37, 38^. Moreover, some methods consider taking some polynomial and rational functions as a prior information which is usually difficult to obtain in advance, for subsequent model selection^39, 40^.

In this paper, we concern over inferring complex nonlinear causal networks from time-series and propose a new method termed as group lasso nonlinear conditional granger causality (GLasso-NCGC). Particularly, we establish a general data-driven framework for nonlinear network reconstruction from time-series with limited observations, which doesn’t require the pre-defined model or some polynomial and rational functions as a prior information. With obtaining multi-source datasets based on the data-fusion strategy introduced in recent high quality paper^3^, we first introduce the formulation of multivariate nonlinear conditional granger causality model (NCGC), and exploit different groups of radial basis functions (RBF) to approximate the nonlinear interactions between each pair of nodes respectively. Then we decompose the task of inferring the whole network into local neighborhood selections centered at each target node. Together with the natural sparsity of real complex networks, group lasso regression^41^ is utilized for each local structure recovery, where different RBF variables from different centers in each node should be either eliminated or selected as a group. Next, we obtain the candidate structure of the network by resolving the problem of group lasso regression. As a result, the final network can be judged by significance levels with Fisher statistic test for removing false existent links.

For the purpose of performance evaluations for our proposed method, simulated datasets from nonlinear vector autoregressive model are firstly generated. Compared with CGC, Lasso-CGC and NCGC, we find GLasso-NCGC outperforms other methods in terms of Area Under Precision-Recall curve (AUPR) and Receiver-Operating-Characteristic curve (AUROC). Effects of data size and noise intensity are also investigated. Besides, the simulation based on nonlinear dynamic models with network topology given in advance is carried out. We consider two classical nonlinear dynamic models which are used for modelling biochemical reaction networks^40^ and gene regulatory networks^42^ respectively. Especially for gene regulatory networks with Michaelis-Menten dynamics, we simulate gene regulatory model on random, small-world and scare-free networks. Then we explore the performance of these methods influenced by different average degrees, noise intensities and amounts of data simultaneously for these three types of networks. Based on the sub-challenge of Dialogue on Reverse Engineering Assessment and Methods (DREAM), we finally use the benchmark datasets of DREAM3 Challenge4 for investigation. All of the results demonstrate the proposed method GLasso-NCGC executes best on the previous mentioned datasets, which is fully certified to be optimal and robust to noise.

## Models and Methods

### Multivariate conditional granger causality

Consider *N* time-course variables {*X*, *Y*, *Z*_1_, *Z*_2_, …, *Z*_*N*−2_} in multivariate conditional granger causality model, the current value of *Y*_*t*_ can be expressed by the past values of *Y* and *Z*_1_, *Z*_2_, …, *Z*_*N*−2_ in Equation (1). Meanwhile, *Y*_*t*_ can be also written as the joint representation of the past values of *Y*, *X* and *Z*_1_, *Z*_2_, …, *Z*_*N*−2_ in Equation (2).

1 $${Y}_{t}=\sum _{i=1}^{p}{a}_{i}{Y}_{t-i}+\sum _{k=1}^{N-2}\sum _{i=1}^{p}{c}_{k,i}{Z}_{k,t-i}+{\varepsilon }_{1}$$

2 $${Y}_{t}=\sum _{i=1}^{p}{a}_{i}^{^{\prime} }{Y}_{t-i}+\sum _{i=1}^{p}{b}_{i}^{^{\prime} }{X}_{t-i}+\sum _{k=1}^{N-2}\sum _{i=1}^{p}{c}_{k,i}^{^{\prime} }{Z}_{k,t-i}+{\varepsilon }_{2}$$

where, ${a}_{i},{c}_{k,i},{a}_{i}^{^{\prime} },{b}_{i}^{^{\prime} },{c}_{k,i}^{^{\prime} }$ are the coefficients in VAR model, *p* is the model order, *ε*_1_ and *ε*_2_ are the noise terms. Let *Z* = {*Z*_1_, *Z*_2_, …, *Z*_*N*−2_}, then the CGC index can be shown as *CGCI*_*X*→*Y*|*Z*_ = ln (var(*ε*_1_)/var(*ε*_2_)), which is used to analyze the conditional causal interaction between two temporal variables. If the variance var(*ε*_1_) is larger than the variance var(*ε*_2_), i.e. *CGCI*_*X*→*Y*|*Z*_ > 0, *X* causes *Y* conditioned on the other sets of *N* − 2 variables *Z*. Generally, the statistic judgement of terminal results can be executed by significant levels with F-test.

### Group lasso nonlinear conditional granger causality

The unified framework of multivariate nonlinear conditional granger causality (NCGC) model is proposed as shown in Equation (3).

3 $${\bf{Y}}={\rm{\Phi }}({\bf{X}}){\bf{A}}+{\bf{E}}$$

where, target variables ${\bf{Y}}=[{{\bf{y}}}_{1}\quad {{\bf{y}}}_{2}\cdots {{\bf{y}}}_{N}]$, coefficient matrix ${\bf{A}}=[{{\boldsymbol{\alpha }}}_{1}\,{{\boldsymbol{\alpha }}}_{2}\cdots {{\boldsymbol{\alpha }}}_{N}]$, noise terms ${\bf{E}}=[{\varepsilon }_{1}\,{\varepsilon }_{2}\cdots {\varepsilon }_{{\boldsymbol{N}}}]$, **Φ**(**X**) is data matrix of nonlinear kernel functions. The detailed descriptions for all the elements above are given in Equation (4).

For each target variable **y**_*i*_ ∈ **Y**, we can obtain *N* independent equations as follows.

4 $${{\bf{y}}}_{i}={\rm{\Phi }}({\bf{X}}){\alpha }_{i}+{\varepsilon }_{i},\quad i=\mathrm{1,2,}\ldots ,N$$

where,

$$\begin{array}{ccc}{{\bf{y}}}_{i} & = & {[\begin{array}{cccc}{x}_{i}(p+1) & {x}_{i}(p+2) & \cdots  & {x}_{i}(T)\end{array}]}^{{\bf{T}}}\\ {\rm{\Phi }}({\bf{X}}) & = & [\begin{array}{cccc}{\rm{\Phi }}({{\bf{X}}}_{1}) & {\rm{\Phi }}({{\bf{X}}}_{2}) & \cdots  & {\rm{\Phi }}({{\bf{X}}}_{N})\end{array}]\\ {{\bf{X}}}_{j} & = & [\begin{array}{c}{{\bf{X}}}_{j}^{1}\\ {{\bf{X}}}_{j}^{2}\\ \vdots \\ {{\bf{X}}}_{j}^{T-p}\end{array}]=[\begin{array}{cccc}{x}_{j}(p) & {x}_{j}(p-1) & \cdots  & {x}_{j}(1)\\ {x}_{j}(p+1) & {x}_{j}(p) & \cdots  & {x}_{j}(2)\\ \vdots  & \vdots  & \ddots  & \vdots \\ {x}_{j}(T-1) & {x}_{j}(T-2) & \cdots  & {x}_{j}(T-p)\end{array}]\\ {\rm{\Phi }}({{\bf{X}}}_{j}^{k}) & = & [\begin{array}{cccc}{\phi }_{1}({{\bf{X}}}_{j}^{k}) & {\phi }_{2}({{\bf{X}}}_{j}^{k}) & \cdots  & {\phi }_{n}({{\bf{X}}}_{j}^{k})\end{array}],\\ k & = & 1,2,\ldots ,t-p\\ {\phi }_{p}({{\bf{X}}}_{j}^{k}) & = & \exp (-{\parallel {{\bf{X}}}_{j}^{k}-{\mathop{{\bf{X}}}\limits^{ \sim }}_{j}^{\rho }\parallel }^{2}/2{\sigma }^{2}),\\ \rho  & = & 1,2,\ldots ,n\\ {\alpha }_{i} & = & {[\begin{array}{cccc}{\alpha }_{i1} & {\alpha }_{i2} & \ldots  & {\alpha }_{iN}\end{array}]}^{{\bf{T}}}\\ {\alpha }_{ij} & = & {[\begin{array}{cccc}{a}_{ij}(1) & {a}_{ij}(2) & \ldots  & {a}_{ij}(n)\end{array}]}^{{\bf{T}}},\\ j & = & 1,2,\ldots ,N\end{array}$$

*N* is the number of variables (time-series), *T* is the length of each time-series. The value of *i*-th variable *x*_*i*_(*t*) can be observed at time *t*. Here, the form of ${\phi }_{\rho }({{\bf{X}}}_{j}^{k})$ is taken as radial basis function. ${\{{\tilde{{\bf{X}}}}_{j}^{\rho }\}}_{\rho =1}^{n}$ is the set of *n* centers in the space of **X**_*j*_, which can be acquired by *k*-means clustering methods. *α*_*i*_ is the coefficient between target variable **y**_*i*_ and **Φ**(**X**). *a*_*ij*_(*n*) is the coefficient corresponding to the function ${\phi }_{n}({{\bf{X}}}_{j}^{k})$.

Generally, we can use least square method to deal with Equation (4). But the solution procedure of Equation (4) may be problematic when the number of variables is relatively larger than the number of available samples. With the knowledge of sparsity in **A**, we can turn to regularization-based methods for help. In this case, it’s noteworthy that apparent groupings exist among variables. So variables belonging to the same group should be regarded as a whole. Here, a series of RBF variables from the different centers in the same time-series should be either eliminated or selected as a group. Then group lasso is adopted to solve this problem of sparse regression as follows.

5 $${\widehat{\alpha }}_{i}=\mathop{{\rm{argmin}}}\limits_{{\alpha }_{i}}({\Vert {{\bf{y}}}_{i}-{\rm{\Phi }}({\bf{X}}){\alpha }_{i}\Vert }_{2}^{2}+{\lambda }_{i}\sum _{j=1}^{N}{\Vert {\alpha }_{ij}\Vert }_{2})$$

In Equation (5), we take *l*_2_ norm as the intra-group penalty.

The detailed algorithm of Group Lasso Nonlinear Conditional Granger Causality is shown in Algorithm 1.

With small samples at hand, first-order vector autoregressive model is usually considered^20, 21^. Besides, in our consideration, model order is also set as *p* = 1, which is in accordance with the characteristics of dynamic systems governed by state-space equations. For the time-delayed dynamic systems, the implementation of higher-order vector autoregressive model can be straightly extended. In addition, we can use cross validation criterion for the selection of optimal parameters, such as the number of RBF centers *n* and the coefficient of penalty terms *λ*_*i*_. Then we mainly describe the selection of *λ*_*i*_ as follows. We use two stages of refined selection which are similarly adopted by Khan *et al*.^43^. In the first stage, we set the coarse values of the search space *λ* ∈ {*λ*_1_, *λ*_2_, *λ*_3_, *λ*_4_, *λ*_5_}, which determines the neighborhood of the optimal *λ*_*i*_. In the second stage, we obtain the neighborhood of the optimal *λ*_*i*_ for refined search, i.e. *λ* ∈ [0.5*λ*_*i*_, 1.5*λ*_*i*_], the interval Δ*λ* = *kλ*_*i*_ (0 < *k* < 1). Here, *λ*_1_ = 10^−4^, *λ*_2_ = 10^−3^, *λ*_3_ = 10^−2^, *λ*_4_ = 10^−1^, *λ*_5_ = 1, *k* = 0.1. For example, if we choose *λ*_3_ = 10^−2^ in the first stage, we next confine the refined search in the range of *λ* ∈ [0.005, 0.015], where Δ*λ* = 10^−3^. For large-scale networks, we can just take relatively larger *k* to reduce the range of search space and ensure the low computational cost.

Meanwhile, in order to ensure the variety of datasets, multiple measurements are often carried out under different conditions (adding perturbations or knocking out nodes *et al*.). As a result, the extension of Equation (3) can be written as the following formula.

6 $$[\begin{array}{c}{\tilde{{\bf{Y}}}}_{1}\\ \vdots \\ {\tilde{{\bf{Y}}}}_{m}\end{array}]={\rm{\Phi }}([\begin{array}{c}{\tilde{{\bf{X}}}}_{1}\\ \vdots \\ {\tilde{{\bf{X}}}}_{m}\end{array}]){\bf{A}}+[\begin{array}{c}{\tilde{{\bf{E}}}}_{1}\\ \vdots \\ {\tilde{{\bf{E}}}}_{m}\end{array}]$$

In Equation (6), *m* denotes the number of measurements. At *m*-th measurement, ${\tilde{{\bf{Y}}}}_{m}$ and ${\tilde{{\bf{X}}}}_{m}$ are acquired for integrating them in Equation (6). Then Equation (6) can be also divided into *N* independent equations that would be solved by group lasso optimization with Equation (5). Finally, we execute nonlinear conditional granger causality with F-test in terms of the given significant level *P*_*val*_. In our paper, we set *P*_*val*_

**Table:** 

**Algorithm 1** Group Lasso Nonlinear Conditional Granger Causality
1: **Input:** Time-series data {*x*_*i*_(*t*)}, *i* = 1, 2, …, *N*; *t* = 1, 2, …, *T*. *N* is the number of variables. *T* is the length of each time-series.
2: According to model order *p*, form the data matrix **X** = [**X**_1_, **X**_2_, …, **X**_*N*_] and target matrix **Y** = [**y**_1_, **y**_2_, …, **y**_*N*_].
3: Formulize the data matrix **X** into **Φ**(**X**) based on radial basis functions as shown in Equation (4).
4: **For** each target variable **y**_*i*_ ∈ **Y**
**•** Execute group lasso:
$${\tilde{\alpha }}_{i}=\mathop{{\rm{a}}{\rm{r}}{\rm{g}}{\rm{m}}{\rm{i}}{\rm{n}}}\limits_{{\alpha }_{i}}({\Vert {{\rm{y}}}_{i}-{\rm{\Phi }}({\bf{X}}){\alpha }_{i}\Vert }_{2}^{2}+{\lambda }_{i}\sum _{j=1}^{N}{\Vert {\alpha }_{ij}\Vert }_{2}).$$
**•** Obtain candidate variable sets *S*_*i*_ for each **y**_*i*_ according to ${\hat{\alpha }}_{i}$. Rearrange data matrix **Φ**(**X**) with *S*_*i*_ expressed as ${{\rm{\Phi }}}_{{S}_{i}}$ and reform the expression of nonlinear conditional granger causality model.
**•** Execute nonlinear conditional granger causality with F-test in terms of the given significant level *P*_*val*_. Confirm the causal variables of **y**_*i*_.
** end**
5: **Output:** Causal interactions among *N* variables, i.e. adjacency matrix ${{\mathbb{R}}}^{N\times N}$.

= 0.01 for all the statistic analysis.

## Results

### Nonlinear vector autoregressive model

The first simulation is based on a nonlinear vector autoregressive model with *N* = 10 nodes (variables), see Equation (7).

7 $$\begin{array}{rcl}{x}_{\mathrm{1,}t} & = & 0.5{x}_{\mathrm{1,}t-1}+{\varepsilon }_{\mathrm{1,}t}\\ {x}_{\mathrm{2,}t} & = & 0.6\,\cos \,({x}_{\mathrm{2,}t-1})+{\varepsilon }_{\mathrm{2,}t}\\ {x}_{\mathrm{3,}t} & = & 0.7{e}^{-{x}_{\mathrm{3,}t-1}^{2}/2}+{\varepsilon }_{\mathrm{3,}t}\\ {x}_{\mathrm{4,}t} & = & 0.8{x}_{\mathrm{7,}t-1}+{\varepsilon }_{\mathrm{4,}t}\\ {x}_{\mathrm{5,}t} & = & 0.9{x}_{\mathrm{8,}t-1}+{\varepsilon }_{\mathrm{5,}t}\\ {x}_{\mathrm{6,}t} & = & \sin \,({x}_{\mathrm{1,}t-1})-0.6{x}_{\mathrm{9,}t-1}^{2}+{\varepsilon }_{\mathrm{6,}t}\\ {x}_{\mathrm{7,}t} & = & 2\,\cos \,({x}_{\mathrm{2,}t-1})+0.6\,\sin \,({x}_{\mathrm{10,}t-1})+{\varepsilon }_{\mathrm{7,}t}\\ {x}_{\mathrm{8,}t} & = & 0.8\,\cos \,({x}_{\mathrm{3,}t-1})+\,\cos \,({x}_{\mathrm{6,}t-1})+1+{\varepsilon }_{\mathrm{8,}t}\\ {x}_{\mathrm{9,}t} & = & \sin \,({x}_{\mathrm{4,}t-1})-0.8{x}_{\mathrm{7,}t-1}+{\varepsilon }_{\mathrm{9,}t}\\ {x}_{\mathrm{10,}t} & = & {e}^{-{x}_{\mathrm{8,}t-1}^{2}\mathrm{/2}}+{\varepsilon }_{\mathrm{10,}t}\end{array}$$

**Figure 1:**
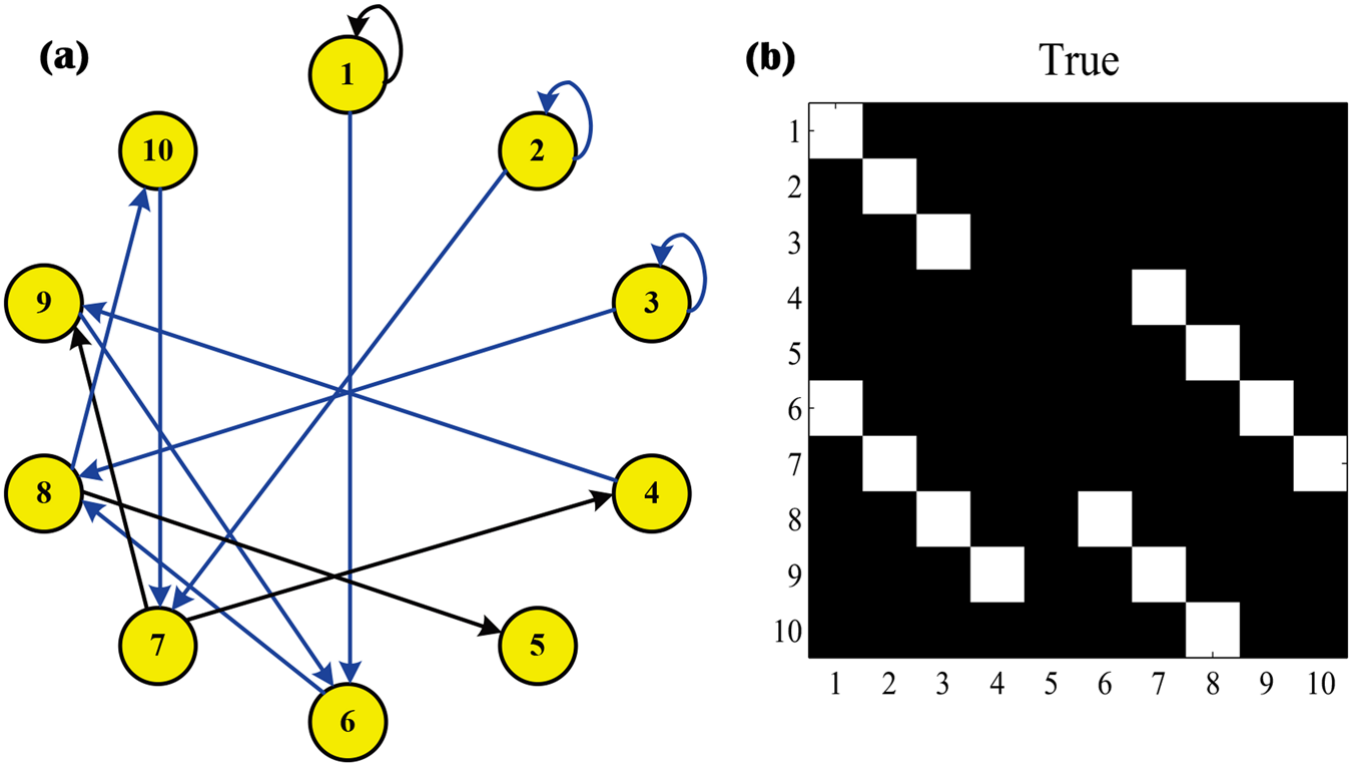
Directed graph and true adjacency matrix of nonlinear VAR model.

The directed graph from Equation (7) together with true adjacency matrix are shown in Fig. 1. In Fig. 1(a), black lines are linear edges and blue lines are nonlinear edges. In Fig. 1(b), white points are existent edges among nodes, while black areas stand for nonexistent edges. Next, the synthetic datasets (*M* = 100 samples) are obtained based on the model of Equation (7), where *ε* is zero-mean uncorrelated gaussian noise terms with identical unit variance.

**Figure 2:**
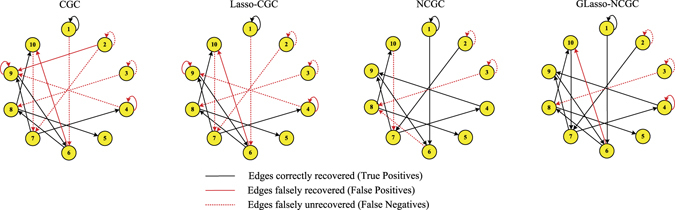
Networks inferred by CGC, Lasso-CGC, NCGC, GLasso-NCGC in nonlinear VAR simulation.

Networks inferred by CGC, Lasso-CGC, NCGC and GLasso-NCGC are shown in Fig. 2. According to the number of edges correctly recovered (True Positives), we can find that GLasso-NCGC almost captures all the existing edges except for the edges 2 → 2, 3 → 3, 3 → 8. Compared with GLasso-NCGC, NCGC additionally fails to recover two existing edges 6 → 8, 10 → 7. Due to neglect the nonlinear influences among nodes, both CGC and Lasso-CGC fail to find many existing edges, which leads to many edges falsely unrecovered (False Negatives).

**Figure 3:**
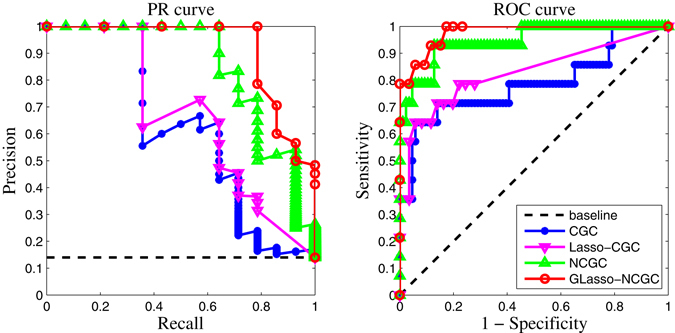
PR curves and ROC curves of CGC, Lasso-CGC, NCGC, GLasso-NCGC in nonlinear VAR simulation.

For further measuring performance we plot ROC curves and PR curves. Figure 3 is PR and ROC curves generated by CGC, Lasso-CGC, NCGC, GLasso-NCGC respectively. Obviously, it can be seen from Fig. 3 that GLasso-NCGC outperforms its competitors with the highest score (AUROC and AUPR).

**Figure 4:**
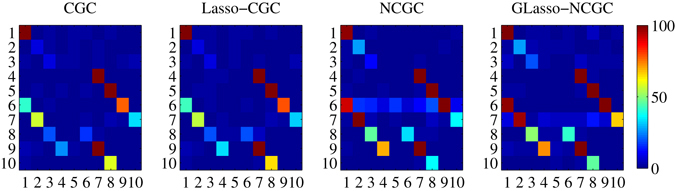
Discovery rate matrixes inferred from 100 realizations in nonlinear VAR simulation.

In the following section, the performance of robust estimation with different methods is explored. Firstly, multi-realizations are carried out, here the number of realizations is set as 100. Figure 4 demonstrates discovery rate matrixes from multi-realizations. Table 1 shows the comparison of discovery rate of true positives inferred by these methods. Discovery rate means the total number of each edge rightly discovered over multi-realizations. Compared with CGC, Table 1 summarizes Lasso-CGC improves the performance with relatively larger discovery rate in true edges. However, due to neglecting the nonlinearity of modeling, they can’t manifest better performance than NCGC and GLasso-NCGC. In general, for nonlinear edges, we can discover GLasso-NCGC nearly outperforms other methods. Specially, for edges 1 → 6, 2 → 7, 3 → 8 and 4 → 9, GLasso-NCGC and NCGC greatly identify these true causal edges at large percentages of 100 independent realizations. For linear edges, both GLasso-NCGC and NCGC also maintain high discovery rate.

**Table 1:** Comparison of discovery rate of true positives extracted from true adjacency matrix of nonlinear VAR model.

		CGC	Lasso-CGC	NCGC	GLasso-NCGC
Nonlinear	1 → 6	41%	43%	92%	97%
	2 → 2	8%	9%	28%	28%
	2 → 7	57%	56%	100%	96%
	3 → 3	9%	9%	11%	19%
	3 → 8	20%	20%	46%	50%
	4 → 9	26%	34%	69%	73%
	6 → 8	16%	19%	33%	42%
	8 → 10	58%	65%	37%	46%
	9 → 6	78%	79%	100%	100%
	10 → 7	33%	32%	36%	66%
Linear	1 → 1	99%	99%	97%	99%
	7 → 4	100%	100%	100%	100%
	7 → 9	100%	100%	100%	100%
	8 → 5	100%	100%	100%	100%

Relatively, GLasso-NCGC utilizes group lasso to promote the performance of NCGC by silencing many false positives and realize the best reconstruction of adjacency matrix at last. Average AUROC and Average AUPR of different methods over 100 realizations are calculated in Table 2.

**Table 2:** Average AUPR and Average AUROC of different methods in nonlinear VAR simulation (standard deviation (SD) in parentheses).

Methods	Aver-AUROC (SD)	Aver-AUPR (SD)
CGC	0.8830 (0.0477)	0.6810 (0.0751)
Lasso-CGC	0.8837 (0.0448)	0.6967 (0.0673)
NCGC	0.9301 (0.0398)	0.7589 (0.0621)
GLasso-NCGC	**0**.**9519** (0.0356)	**0**.**8044** (0.0587)

The values are obtained by averaging over 100 independent realizations.

**Figure 5:**
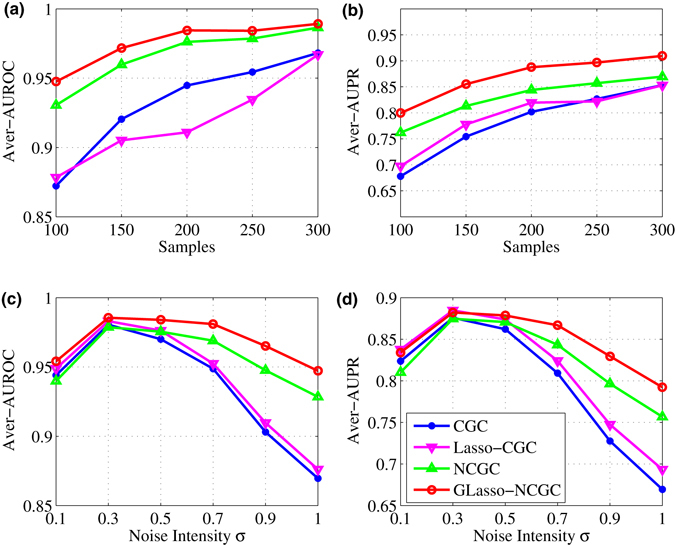
Simulation of nonlinear VAR model. Average AUROC and Average AUPR of different methods acrossing samples (100~300), noise intensities *σ* (0.1~1) respectively. The values of points in these figures are computed by averaging over 100 independent realizations. (**a**,**b**) with noise ${\mathscr{N}}\mathrm{(0,1)}$, while (**c**,**d**) with samples *M* = 100.

Then, we explore the performance of CGC, Lasso-CGC, NCGC and GLasso-NCGC influenced by different number of samples. Given that the number of samples varies from 100 to 300, at each point, we get the Average AUROC and Average AUPR over 100 independent realizations respectively. From Fig. 5(a) and (b), we find the performance of these four methods improves as the number of samples increases. GLasso-NCGC scores highest in both Average AUROC and Average AUPR.

Next, given samples with *M* = 100, the performance of CGC, Lasso-CGC, NCGC and GLasso-NCGC influenced by different noise intensities *σ* (0.1~1) is also compared as shown in Fig. 5(c) and (d). At each intensity of noise, the Average AUROC and Average AUPR over 100 independent realizations are computed respectively. GLasso-NCGC also wins the best score in both Average AUROC and Average AUPR.

### Nonlinear dynamic model

Consider the complex dynamic system of *N* variables (nodes) with the following coupled nonlinear equations.

8 $${\dot{x}}_{i}=F({x}_{i})+\sum _{j=\mathrm{1,}j\ne i}^{N}{b}_{ij}H({x}_{i},{x}_{j})+{\varepsilon }_{i}$$

where state vector **x** = [*x*_1_, *x*_2_, …, *x*_*N*_]^*T*^, the first term on the right-hand side of Equation (8) describes the self-dynamics of the *i*-th variable, while the second term describes the interactions between variable *i* and its interacting partners *j*. The nonlinear functions *F*(*x*_*i*_) and *H*(*x*_*i*_, *x*_*j*_) represent the dynamical laws that govern the variables of system. Let **B** be the adjacent matrix which describes the interactions among variables. *b*_*ij*_ ≠ 0 if the *j*-th variable connects to the *i*-th one; otherwise, *b*_*ij*_ = 0. *ε*_*i*_ is zero-mean uncorrelated gaussian noise with variance *σ* ^2^. Indeed, these is no unified manner to establish the nonlinear framework for all the complex networked systems. But Equation (8) has the broad applications in many science domains. With an appropriate choice of *F*(*x*_*i*_) and *H*(*x*_*i*_, *x*_*j*_), Equation (8) is used to model various known systems, ranging from ecological systems, social systems and physical systems^11, 18, 40, 42^. Specifically, Equation (8) can be transformed into discrete-time expressions.

$${x}_{i}({t}_{k+1})={x}_{i}({t}_{k})+F({x}_{i}({t}_{k}))+\sum _{j=\mathrm{1,}j\ne i}^{N}{b}_{ij}H({x}_{i}({t}_{k}),{x}_{j}({t}_{k}))+{\varepsilon }_{i}({t}_{k})$$

where, *t*_*k*+1_ − *t*_*k*_ = 1. Here, the simulation systems are as follows.

**S1:** Biochemical reaction network (BRN).

9 $$\begin{array}{ccc}{\dot{x}}_{1} & = & -{\gamma }_{1}{x}_{1}+\frac{{\alpha }_{1}}{1+{x}_{6}^{{n}_{1}}}+{\varepsilon }_{1}\\ {\dot{x}}_{2} & = & -{\gamma }_{2}{x}_{2}+\frac{{\alpha }_{2}}{1+{x}_{4}^{{n}_{2}}}+{\varepsilon }_{2}\\ {\dot{x}}_{3} & = & -{\gamma }_{3}{x}_{3}+\frac{{\alpha }_{3}}{1+{x}_{5}^{{n}_{3}}}+{\varepsilon }_{3}\\ {\dot{x}}_{4} & = & -{\gamma }_{4}{x}_{4}+{\beta }_{1}{x}_{1}+{\varepsilon }_{4}\\ {\dot{x}}_{5} & = & -{\gamma }_{5}{x}_{5}+{\beta }_{2}{x}_{2}+{\varepsilon }_{5}\\ {\dot{x}}_{6} & = & -{\gamma }_{6}{x}_{6}+{\beta }_{3}{x}_{3}+{\varepsilon }_{6}\end{array}$$

Here, *x*_1_, *x*_2_ and *x*_3_ are concentrations of the mRNA transcripts of genes 1,2,3 respectively; *x*_4_, *x*_5_ and *x*_6_ are concentrations of the proteins of genes 1,2,3 respectively; *α*_1_, *α*_2_, *α*_3_ are maximum promoter strength for the corresponding gene; *γ*_1_, *γ*_2_, *γ*_3_ are mRNA decay rates; *γ*_4_, *γ*_5_, *γ*_6_ are protein decay rates; *β*_1_, *β*_2_, *β*_3_ are protein production rates; *n*_1_, *n*_2_, *n*_3_ are hill coefficients.

The parameters of Equation (9) are given as follows. *α*_1_ = 4, *α*_2_ = 3, *α*_3_ = 5; *γ*_1_ = 0.3, *γ*_2_ = 0.4, *γ*_3_ = 0.5; *γ*_4_ = 0.2, *γ*_5_ = 0.4, *γ*_6_ = 0.6; *β*_1_ = 1.4, *β*_2_ = 1.5, *β*_3_ = 1.6; *n*_1_ = *n*_2_ = *n*_3_ = 4. Meanwhile, we set the number of samples *M* = 50 and noise $\varepsilon \sim {\mathscr{N}}\,{\mathrm{(0,0.1}}^{2})$.

**Figure 6:**
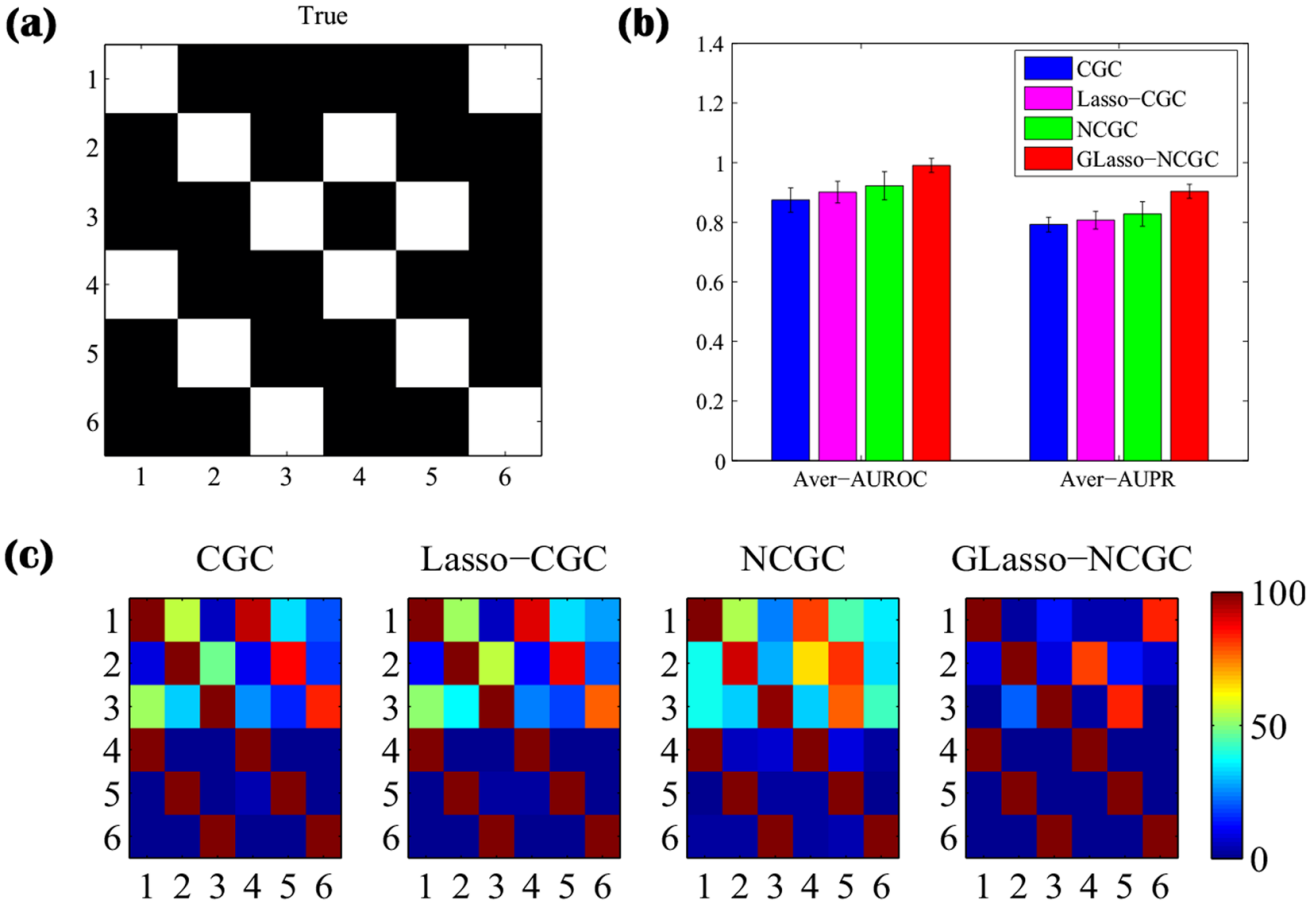
Simulation of BRN. The number of multi-realizations is set as 100. (**a**) True network. (**b**) Average AUROC and Average AUPR with standard deviation over multi-realizations. (**c**) Discovery rate matrixes inferred from multi-realizations.

Based on the true network [Fig. 6(a)] derived from Equation (9), we can find GLasso-NCGC almost recover all of the real edges over 100 independent realizations as shown in Fig. 6(c). From Fig. 6(c), CGC, Lasso-CGC and NCGC can’t discover the entire true positives but result in lots of false positives. In detail, we next choose some representative edges for further analysis. The discovery rate of these representative edges are calculated in Table 3. Compared with CGC, Lasso-CGC and NCGC, GLasso-NCGC demonstrates a considerable advantage for both these true positives and false positives.

**Table 3:** Comparison of discovery rate of representative edges extracted from true adjacency matrix in BRN simulation.

		CGC	Lasso-CGC	NCGC	GLasso-NCGC
True Positives	6 → 1	20%	27%	36%	83%
	4 → 2	10%	12%	65%	80%
	5 → 3	15%	18%	78%	83%
False Positives	4 → 1	93%	90%	81%	4%
	5 → 2	86%	88%	82%	13%
	6 → 3	84%	78%	44%	1%

With the quantitative comparison of these methods in Fig. 6(b), we can observe GLasso-NCGC get the highest Average AUROC and Average AUPR with the smallest standard deviations (resp. 0.9905 (0.0234) and 0.9037 (0.0235)).

**Figure 7:**
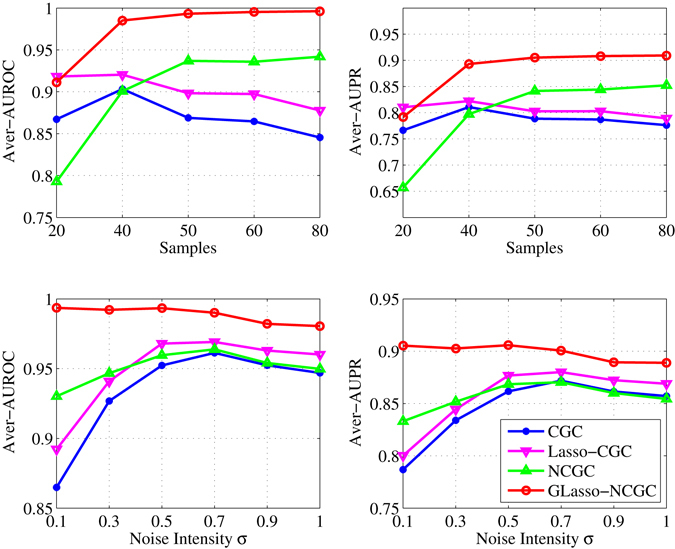
Simulation of BRN. Average AUROC and Average AUPR acrossing samples (20~80), noise intensities *σ* (0.1~1) respectively. The values of points in these figures are computed by averaging over 100 independent realizations.

Here, we also explore the performance of robust estimation with different Granger Causality methods. Figure 7 demonstrates the curves of Average AUROC and Average AUPR of different methods acrossing samples (20~80) and noise intensities *σ* (0.1~1) respectively. The values are obtained by averaging over 100 independent realizations.

**S2:** Gene Regulatory Networks (GRN).

Suppose that gene interactions can be modeled by Michaelis-Menten equation as follows.

10 $${\dot{x}}_{i}=-{x}_{i}^{f}+\sum _{j=\mathrm{1,}j\ne i}^{N}{b}_{ij}\frac{{x}_{j}^{h}}{{x}_{j}^{h}+1}+{\varepsilon }_{i}$$

where, *f* = 1, *h* = 2.

We first construct adjacent matrix **B** with *N* nodes and simulate gene regulatory model with three classical types of networks, including random, small-world and scare-free networks (resp. RN, SW, SF). Then synthetic datasets of *M* samples are generated from the gene regulatory model of Equation (10). In order to reconstruct large-scale complex networks, multiple measurements from different conditions should be executed by adding perturbations in some different ways or with random initial values. Meanwhile, the outputs of model are supposed to be contaminated by gaussian noise. The number of multiple measurements is set as *m*. *T* time points are obtained at each measurement. As a result, data matrix with *M* × *N* is collected (*M* = *mT*).

**Figure 8:**
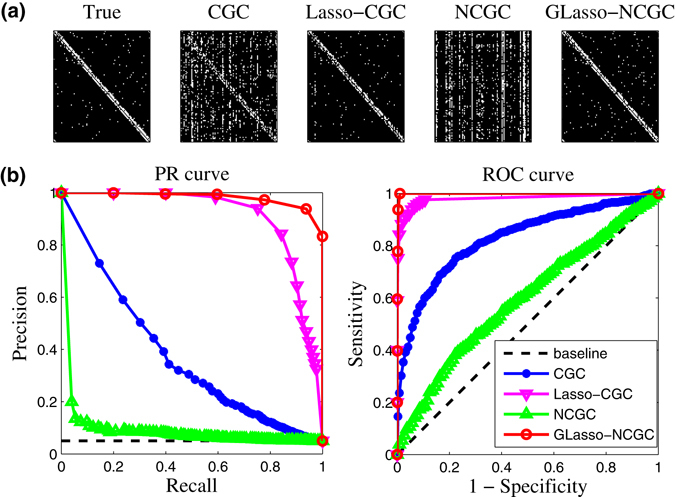
Simulation of GRN model on SW network (*N* = 100, $\langle k\rangle$ = 5, *M* = 400). (**a**) True network and reconstructed network inferred by different methods with noise ${\mathscr{N}}{\mathrm{(0,0.1}}^{2})$. (**b**) PR curves and ROC curves with noise ${\mathscr{N}}{\mathrm{(0,0.1}}^{2})$.

**Figure 9:**
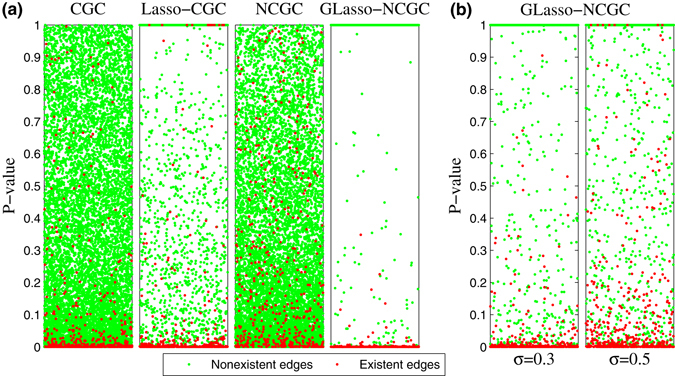
Simulation of GRN model on SW network (*N* = 100, $\langle k\rangle$ = 5, *M* = 400). (**a**) Reconstructed values of elements in different inferred matrixes with noise ${\mathscr{N}}{\mathrm{(0,0.1}}^{2})$. (**b**) Reconstructed values of elements in recovered matrixes by GLasso-NCGC with noise ${\mathscr{N}}{\mathrm{(0,0.3}}^{2})$ and ${\mathscr{N}}{\mathrm{(0,0.5}}^{2})$ respectively.

Here, we take size 100 SW network with average degree 〈*k*〉 = 5 for detailed analysis. We set gaussian noise intensity as 0.1 and generated 400 samples with 100 multi-measurements (each measurement with 4 time points). True SW network are given in Fig. 8(a) together with reconstructed networks which are inferred by different methods. From Fig. 8(a), we can observe that the network inferred by GLasso-NCGC is most similar to the true network. To some extent, both CGC and Lasso-CGC have similar structures compared with the true network. However, they generated lots of false positives. Meanwhile, it can be seen that NCGC almost failed in this case. Because there are more parameters involved in NCGC model and the ratio of the number of samples to the number of parameters is relatively smaller than that of CGC model. Actually, the best performance of GLasso-NCGC proves that regularization-based methods have superiority in the case of more parameters and relatively smaller samples. In Fig. 8(b), we plot PR curves and ROC curves of CGC, Lasso-CGC, NCGC and GLasso-NCGC. The almost perfect reconstruction is ensured by GLasso-NCGC. Furthermore, reconstructed values of elements in different inferred matrixes for size 100 SW network can be shown in Fig. 9(a). Red points are existent edges and green points are nonexistent edges. CGC, Lasso-CGC and NCGC cannot make a distinction between existent and nonexistent edges, because there are so many overlaps between red and green points without a certain threshold (*P*_*val*_) for separation. However, GLasso-NCGC shows a vast and clear gap between existent and nonexistent edges. Based on the consideration of robustness, we next apply our method with respect to different intensities of noise ${\mathscr{N}}\,{\mathrm{(0,0.3}}^{2})$ and ${\mathscr{N}}\,{\mathrm{(0,0.5}}^{2})$ respectively. From Fig. 9(b), GLasso-NCGC also maintains a relatively good performance with strong measurement noise.

Then we explore the performance of these methods influenced by different average degrees, noise intensities and amounts of data simultaneously for different types of networks. The detailed results are shown in Table 4. In general, all of the results demonstrate the optimality and robustness of GLasso-NCGC.

**Table 4:** The Average AUROC and Average AUPR with standard deviation in parentheses of different methods based on three types of networks, random (RN), small-world (SW) and scare-free (SF).

Type	*N*	〈*k*〉	*σ*	*M*	CGC	Lasso-CGC	NCGC	GLasso-NCGC
					Aver-AUROC/Aver-AUPR (SD/SD)			
RN	100	5	0.1	400	0.6367/0.2257	0.8118/0.6441	0.5472/0.1369	**0**.**9315/0**.**7719**
					(0.0290/0.0395)	(0.0072/0.0181)	(0.0112/0.0075)	(0.0083/0.0227)
		5	0.1	600	0.8022/0.4947	0.8989/0.7981	0.6321/0.2322	**0**.**9755/0**.**9072**
					(0.0234/0.0405)	(0.0102/0.0213)	(0.0256/0.0317)	(0.0036/0.0128)
		5	0.3	400	0.6518/0.2563	0.7963/0.5994	0.5530/0.1389	**0**.**9186/0**.**7476**
					(0.0180/0.0114)	(0.0129/0.0320)	(0.0132/0.0042)	(0.0052/0.0102)
		8	0.1	400	0.7394/0.3308	0.9281/0.8382	0.5966/0.1341	**0**.**9714/0**.**8967**
					(0.0233/0.0319)	(0.0104/0.0162)	(0.0192/0.0188)	(0.0034/0.0150)
SW	100	5	0.1	400	0.8648/0.4658	0.9769/0.8823	0.6065/0.0929	**0**.**9996/0**.**9895**
					(0.0213/0.0503)	(0.0044/0.0142)	(0.0185/0.0140)	(0.0001/0.0034)
		5	0.1	600	0.8895/0.5384	0.9716/0.8826	0.6949/0.1684	**0**.**9997/0**.**9919**
					(0.0311/0.0733)	(0.0070/0.0275)	(0.0279/0.0255)	(0.0001/0.0027)
		5	0.3	400	0.8527/0.4206	0.9643/0.8342	0.6072/0.0894	**0**.**9975/0**.**9570**
					(0.0145/0.0312)	(0.0073/0.0243)	(0.0113/0.0093)	(0.0004/0.0085)
		8	0.1	400	0.6500/0.1970	0.9538/0.8791	0.5476/0.1030	**0**.**9950/0**.**9312**
					(0.0325/0.0309)	(0.0082/0.0196)	(0.0180/0.0081)	(0.0012/0.0118)
SF	100	5	0.1	400	0.8304/0.4703	0.9358/0.8377	0.7680/0.2798	**0**.**9492/0**.**8812**
					(0.0128/0.0278)	(0.0055/0.0082)	(0.0217/0.0320)	(0.0077/0.0174)
		5	0.1	600	0.9216/0.6986	0.9498/0.8854	0.8939/0.5795	**0**.**9638/0**.**9056**
					(0.0098/0.0183)	(0.0025/0.0047)	(0.0110/0.0515)	(0.0050/0.0112)
		5	0.3	400	0.8464/0.4595	0.9233/0.8026	0.6929/0.1941	**0**.**9443/0**.**8415**
					(0.0198/0.0270)	(0.0057/0.0099)	(0.0166/0.0150)	(0.0078/0.0100)
		8	0.1	400	0.7144/0.3078	0.8322/0.6410	0.6409/0.1786	**0**.**9095/0**.**7848**
					(0.0170/0.0206)	(0.0086/0.0211)	(0.0266/0.0279)	(0.0072/0.0230)

The values are computed by averaging over 10 independent realizations. The results of different methods at different conditions are explored (type, *N*, 〈*k*〉, *σ* and *M*). Here, *N* is the size of network, 〈*k*〉 is average degree of network, *σ* is gaussian noise intensity, *M* is the number of samples. The highest scores of the Average AUROC and Average AUPR are highlighted.

In order to compare the computational requirement by these methods, we simulate the SW and SF networks for comparison (*N* = 100, 〈*k*〉 = 5, *M* = 400). And we calculate the average computational time over 10 independent realizations as shown in Table 5. The specifications of the computer used to run the simulations are as follows. Matlab version: R2013a (64 bit); Operating system: Windows 10 (64 bit); Processor: Intel(R) Core(TM) i7-4770 CPU @ 3.40GHZ 3.40GHZ; RAM: 16GB.

**Table 5:** The average computational time (in sec.) of different methods over 10 independent realizations in the simulation of GRN model on SW and SF networks.

Types	CGC	Lasso-CGC	NCGC	GLasso-NCGC
SW	12.7196	4.9657	30.0955	5.4432
SF	12.6883	4.0068	29.0900	6.0324

### DREAM3 Challenge4

Dialogue on Reverse Engineering Assessment and Methods (DREAM) projects establish the general framework for the verification of various algorithms, which have the broad applications in many areas of research (http://dreamchallenges.org/). There are so many challenges in DREAM projects. Here, DREAM3 Challenge4 is used for the further assessment. The aim of DREAM3 Challenge4 is to infer gene regulatory networks with multiple types of datasets.

DREAM3 Challenge4 has three sub-challenges corresponding to gene regulatory networks with size 10, size 50 and size 100 nodes respectively. In our work, time-series of size 50 and size 100 are used, together with their gold standard networks. There are five sub-networks of Yeast or *E. coli*, all of which are with size 50 and size 100 respectively. Meanwhile, time-series datasets of multiple measurements are acquired under some different conditions. For the networks of size 50 and size 100, the number of measurements are set as 23 and 46 respectively (each measurement with 21 time points).

**Figure 10:**
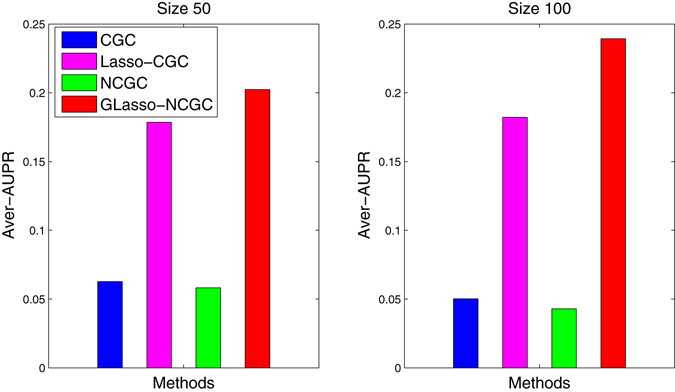
Average AUPR of different methods based on DREAM3 Challenge4 In-Silico networks with Size 50 and Size 100.

To assess these results of different methods, we also calculate the AUROC and AUPR. We can discover that some methods get the highest AUROCs, while their AUPRs are pretty small. In many cases, good AUROC might accompany by a low precision because of a large ratio of FP/TP. Thus, AUPR is taken as the final evaluation metric. In Table 6, GLasso-NCGC gets the best AUPR in all the datasets only except for size 50 network of Yeast1. Furthermore, the average AUPRs of these methods are subsequently computed and plotted as shown in Fig. 10. Finally, GLasso-NCGC is also found to execute optimally with the highest average AUPR.

**Table 6:** Comparison of different methods on DREAM3 Challenge4 In-Silico networks with Size 50 and Size 100.

	Methods	Ecoil1	Ecoil2	Yeast1	Yeast2	Yeast3
	AUROC/AUPR					
Size 50	CGC	0.5180/0.0290	0.5249/0.0388	0.6208/0.0645	0.5285/0.0701	0.5759/0.1115
	Lasso-CGC	0.5643/0.1564	0.5394/0.1060	0.5684/**0**.**2084**	0.5286/0.1654	0.5470/0.2565
	NCGC	0.5492/0.0275	0.5590/0.0437	0.5868/0.0519	0.5227/0.0720	0.5357/0.0961
	GLasso-NCGC	0.5665/**0**.**1686**	0.5316/**0**.**1351**	0.5766/0.2057	0.5272/**0**.**2299**	0.5337/**0**.**2728**
Size 100	CGC	0.5864/0.0335	0.5730/0.0242	0.6196/0.0652	0.5234/0.0615	0.5474/0.0664
	Lasso-CGC	0.5936/0.1660	0.5841/0.1920	0.5807/0.2283	0.5270/0.1757	0.5162/0.1488
	NCGC	0.5629/0.0197	0.5266/0.0179	0.6132/0.0537	0.5348/0.0553	0.5239/0.0681
	GLasso-NCGC	0.5732/**0**.**2016**	0.5696/**0**.**2192**	0.5532/**0**.**2953**	0.5254/**0**.**2438**	0.5116/**0**.**2370**

## Conclusions

Reconstructing complex network is greatly useful for us to analyze and master the collective dynamics of interacting nodes. In our work, with getting multi-source datasets based on the data-fusion strategy, the new method namely group lasso nonlinear coditional granger causality (GLasso-NCGC) is proposed for network recovery with time-series. The evaluations of performance address that GLasso-NCGC is superior to other mentioned methods. Effects of data size and noise intensity are also discussed. Although the models or applications we mainly focus on here are biochemical reaction network and gene regulatory network, our method can be also used to other complex networked systems, such as kuramoto oscillator network and mutualistic network^40, 42^. Here, it is also important to remember that we just adopt the model of first-order nonlinear conditional granger causality (NCGC), which is in accordance with the characteristics of dynamic systems governed by state-space equations. For the time-delayed dynamic systems, such as coupled Mackey-Glass system^28^, the framework of our method can be flexibly extended to higher-order NCGC model with group lasso regression, which can be waited for the prospective researches. In the information era with explosive growth of data, our proposed method provides a general and effective data-driven framework for nonlinear network reconstruction, especially for the complex networked systems that can be turned into the form of **Y** = **Φ**(**X**)**A**.
